# The moderating roles of a “growth mindset” and psychological flexibility on performance anxiety in violin learners

**DOI:** 10.3389/fpsyg.2026.1899477

**Published:** 2026-08-20

**Authors:** Yang Zhou

**Affiliations:** School of Music and Performing Arts, Sichuan University of Arts and Sciences, Dazhou, Sichuan, China

**Keywords:** anxiety coping, gender differences in anxiety, growth mindset, performance anxiety, psychological flexibility, skill learning

## Abstract

**Background:**

Music performance anxiety (MPA), a common situational anxiety subtype, impairs motor execution and stress response in high-stakes violin learning, a context with rigorous technical and evaluative demands. This cross-sectional non-interventional human subject research strictly followed standardized ethical review protocols.

**Objectives:**

This study explored the correlations of growth mindset (GM, cognitive) and psychological flexibility (PF, emotional regulatory) with MPA, examining PF’s moderating effect and gender differences in their anxiety-mitigating pathways.

**Methods:**

A cross-sectional survey of 306 Chinese university violin learners used validated scales to assess GM, PF, and MPA. Data analyses included correlation and hierarchical regression modeling, with gender as a stratification variable. Confirmatory factor analysis (CFA) was additionally conducted to verify construct validity; average variance extracted (AVE), composite reliability (CR), and discriminant validity indices were reported to supplement exploratory factor analysis (EFA) results. Harman’s single-factor test was performed to statistically detect common method bias.

**Results:**

GM and PF both negatively correlated with MPA (*r* = −0.574, *p* < 0.01; *r* = −0.515, *p* < 0.01). PF significantly moderated the GM-MPA relationship, amplifying GM’s anxiety-mitigating effect, and this effect was more pronounced in female learners. After recoding simple slopes and rechecking interaction term centralization procedures, the inconsistent surface coefficient of high PF subgroup was clarified with complete simple slope interpretation and visualized interaction diagram.

**Conclusion:**

GM and PF are key protective factors for situational performance anxiety in skill learning, with a synergistic cognitive-emotional interaction. Findings extend anxiety coping research and provide actionable gender-responsive cognitive-emotional intervention strategies for skill-learning populations. All interpretations are limited to correlational associations rather than causal causal inferences given the cross-sectional design.

## 1 Introduction

Performance anxiety is a common form of situational anxiety that arises in evaluative, goal-directed contexts, characterized by a cluster of negative cognitive (self-doubt, catastrophic thinking), physiological (muscle tension, tachycardia), and behavioral (avoidance, impaired task execution) responses to stress (Chow and Mercado, 2020; Twitchell et al., 2025). Unlike clinical anxiety disorders, situational performance anxiety is triggered by context-specific pressure and is highly prevalent in professional and skill-learning domains (e.g., music, sports, medicine), where successful task execution is contingent on both technical proficiency and adaptive stress regulation (Bellinger et al., 2023; Jamieson et al., 2018). Music performance anxiety (MPA) is a well-documented subtype of situational performance anxiety, and violin learning represents a prototypical high-stakes context for MPA development: the instrument’s technical complexity (e.g., bow control, intonation, and stylistic nuance in repertoire interpretation; Chen, 2024), combined with frequent public performance and evaluative scrutiny, creates a chronic stress environment that elevates anxiety susceptibility (Ou and Qin, 2025; Sokoli et al., 2022). In Chinese music education contexts, MPA affects 64% of violin learners, with high anxiety levels correlating with compromised technical performance and reduced stress coping efficacy (Ou and Qin, 2025), long-term impacts on occupational choice and professional development (Maria, 2020), highlighting the need to identify modifiable psychological factors that buffer against situational performance anxiety in this population.

Anxiety coping research has identified two key modifiable psychological constructs that mitigate situational anxiety: cognitive mindset and psychological flexibility (Kashdan et al., 2020; Yeager and Dweck, 2012). Growth mindset (GM) - the belief that abilities can be developed through deliberate practice, feedback, and reflection (Dweck, 2006) - shapes cognitive appraisals of stress and failure, a core process in anxiety regulation (Karampas et al., 2022; Zarrinabadi et al., 2022). Its theoretical counterpart, fixed mindset, refers to the belief that musical talent and instrumental aptitude are innate, unchangeable traits; fixed mindset holders interpret setbacks as permanent proof of insufficient natural talent, which fuels catastrophic worry before performances. Individuals with a GM interpret setbacks as diagnostic feedback rather than signs of inherent inadequacy, reducing catastrophic thinking and the perceived threat of performance failure-two key cognitive drivers of situational anxiety (Rose et al., 2024; Krieger, 2023). In skill-learning contexts, a GM enhances coping efficacy by fostering sustained effort and adaptive strategy adjustment for expressive performance (Meissner, 2021), which are critical for managing stress in high-evaluative settings (Liu, 2025; McPherson et al., 2022).

Psychological flexibility (PF) - the capacity to accept negative emotions and thoughts, maintain goal-directed behavior, and adapt to situational demands (Bond et al., 2011; Doorley et al., 2020) - is a foundational emotional regulatory construct for addressing pre-performance emotions and music performance anxiety (Kaleńska-Rodzaj, 2021; Kot et al., 2024; Viator et al., 2024). Unlike rigid emotion regulation strategies (e.g., avoidance, suppression), PF promotes adaptive engagement with anxiety: individuals with high PF acknowledge negative emotional states without rumination, redirecting attention to task-relevant goals rather than anxiety-related cognitions (Juncos and de Paiva e Pona, 2018; Limeri et al., 2020). In music performance contexts, PF buffers against MPA by reducing experiential avoidance of anxiety and enhancing focus under pressure (Viator et al., 2024), and it has been shown to moderate the relationship between cognitive factors and anxiety in other skill-learning domains (Öztekin et al., 2025).

While prior research has established independent effects of GM and PF on anxiety, few studies have examined their interactive effects in situational performance anxiety contexts, particularly in non-clinical skill-learning populations (Xu and Xu, 2024). Additionally, gender differences in anxiety regulation are well-documented: females exhibit higher susceptibility to situational performance anxiety (Papageorgi et al., 2013; Sokoli et al., 2022) and may derive greater benefit from cognitive and emotional regulatory interventions (Di Battista, 2025), but the gender-specificity of the GM-PF-anxiety pathway remains understudied in skill-learning contexts. This study addresses these gaps by testing a moderated model of anxiety regulation in violin learners, a high-stakes skill-learning population with high MPA prevalence. We integrate cognitive (GM) and emotional regulatory (PF) constructs to examine: (1) the direct negative effects of GM and PF on MPA; (2) the moderating role of PF in the GM-MPA relationship; and (3) gender differences in the strength of the GM-MPA association.

By focusing on the cognitive-emotional interaction in anxiety coping, this study extends situational anxiety research in three key ways: first, it tests the generalizability of GM and PF as anxiety-buffering factors to the music skill-learning context, a understudied non-clinical situational anxiety domain; second, it identifies a synergistic interaction between cognitive and emotional regulation, clarifying how these two processes combine to mitigate performance anxiety; third, it documents gender differences in this anxiety-regulation pathway, providing insights into tailored coping interventions for gender-specific anxiety susceptibility. The findings have implications for anxiety coping research across skill-learning domains (e.g., sports, medicine) and offer evidence-based strategies for reducing situational performance anxiety in high-evaluative contexts.

## 2 Theoretical framework

### 2.1 Theoretical foundation

Situational performance anxiety arises from a threat appraisal of the environment, where individuals perceive evaluative pressure as a threat to their self-worth or task success (Twitchell et al., 2025; Jamieson et al., 2018). This threat appraisal triggers a cascade of cognitive (catastrophic thinking) and physiological (fight-or-flight) responses that impair task execution and stress coping (Bellinger et al., 2023). Anxiety coping research has emphasized the integration of cognitive appraisal and emotional regulation as core processes for reducing threat appraisal and mitigating anxiety (Kashdan et al., 2020; Karampas et al., 2022): cognitive constructs (e.g., GM) shape how individuals interpret stress and failure, while emotional regulatory constructs (e.g., PF) shape how individuals respond to the negative emotions associated with stress.

Growth mindset exerts its anxiety-mitigating effect through three cognitive appraisal pathways that reduce threat appraisal (Yeager and Dweck, 2012; Rose et al., 2024): (1) cognitive reappraisal (framing failure as feedback rather than a threat); (2) sustained strategic effort (fostering adaptive practice that enhances perceived control over the task); and (3) arousal reappraisal (interpreting physiological anxiety as a performance resource rather than a sign of failure). These pathways reduce the cognitive drivers of situational anxiety, but they rely on the ability to engage with negative emotions without rumination or avoidance- a core component of PF (Doorley et al., 2020; Viator et al., 2024).

Psychological flexibility complements GM by enabling adaptive emotional engagement with anxiety (Bond et al., 2011; Juncos and de Paiva e Pona, 2018). High PF individuals accept anxiety as a normal part of the high-evaluative context, rather than engaging in experiential avoidance (e.g., skipping performances, ruminating on failure), which exacerbates anxiety (Kot et al., 2024; Öztekin et al., 2025). This acceptance allows individuals to act on the cognitive insights of GM: for example, a high PF learner with a GM can acknowledge anxiety before a performance and reframe mistakes as growth opportunities, whereas a low PF learner with a GM may be overwhelmed by anxiety and unable to apply this cognitive appraisal (Viator et al., 2024; Rose et al., 2024). Thus, PF is hypothesized to moderate the GM-MPA relationship, amplifying the anxiety-mitigating effect of GM in individuals with high PF.

Finally, gender differences in anxiety regulation are rooted in socio-cultural and cognitive-emotional factors (Di Battista, 2025; Sokoli et al., 2022): females face stronger socio-cultural expectations of “perfect performance” in evaluative contexts, leading to greater self-criticism and catastrophic thinking-cognitive patterns that GM directly counteracts (Papageorgi et al., 2013; Di Battista, 2025). Males, by contrast, exhibit lower susceptibility to MPA and less self-critical thinking, meaning the anxiety-mitigating effect of GM may be less pronounced (Sokoli et al., 2022). Multiple cross-cultural educational psychology studies further confirm gendered attribution bias: female instrumentalists tend to attribute performance flaws to personal incompetence, while male instrumentalists are more likely to attribute errors to external environmental factors (Di Battista, 2025; Papageorgi et al., 2013). This stable attribution gap widens the protective gap of growth mindset between genders, as growth mindset’s core function of reattributing failure to adjustable practice factors delivers larger emotional relief for female learners trapped in self-blame loops. Thus, the negative association between GM and MPA is hypothesized to be stronger in female learners.

### 2.2 Hypotheses

Based on the cognitive-emotional integration framework of anxiety coping, the following hypotheses were tested:

H1: Growth mindset is significantly negatively associated with music performance anxiety (MPA) in violin learners.

H2: Psychological flexibility is significantly negatively associated with MPA in violin learners.

H3: Psychological flexibility moderates the relationship between growth mindset and MPA, such that the negative association between growth mindset and MPA is stronger in learners with higher psychological flexibility.

H4: The negative association between growth mindset and MPA is more pronounced in female violin learners than in male violin learners.

## 3 Materials and methods

### 3.1 Participants

Participants were recruited via multi-stage stratified random sampling [stratified by university type (conservatory vs. comprehensive university), academic grade, and gender] from violin majors at eight Chinese universities across six provinces (e.g., Beijing, Shanghai, Guangdong). Questionnaires were distributed online via Questionnaire Star, a widely used platform for academic surveys in China. Quality control measures included: (1) excluding responses with inconsistent answers to reverse-coded items; (2) removing surveys that failed attention checks (e.g., “Please select “Strongly Agree” for this item”); (3) omitting responses with abnormally short (<5 min) or long (>30 min) completion times; and (4) excluding homogeneous responses (e.g., selecting the same Likert scale option for all items). After these checks, 306 valid samples were retained.

All survey procedures and participant recruitment were reviewed and approved by the Ethics Committee of Sichuan University of Arts and Sciences, approval number ART-2025-041. All procedures fully complied with the Declaration of Helsinki ethical standards for human subject research.

Key demographic and training characteristics of participants are summarized as follows:

Gender: 135 males (44.12%), 171 females (55.88%);

Age: 18 years or younger (18.30%), 19–22 years (34.31%), 23–26 years (28.10%), 27–30 years (13.73%), 31 years or older (5.56%); M age = 22.3 ± 3.1 years;

Education level: undergraduates (56.54%), master’s students (41.18%), doctoral students (2.29%);

Learning experience: <1 year (3.92%), 1–3 years (39.22%), 4–6 years (35.29%), 7–10 years (16.67%), >10 years (4.90%); weekly practice hours: <2 h (22.88%), 2–5 h (17.97%), 6–10 h (20.92%), 11–15 h (22.22%), >15 h (16.01%);

Performance experience: 1–2 public performances in the past year (37.25%), 3–5 performances (30.07%), 6–10 performances (22.55%), >10 performances (10.13%);

Career intention: planning to pursue a music career (64.05%), not planning (12.75%), unsure (23.20%).

All participants provided electronic informed consent prior to completing the survey, and no personally identifiable information (e.g., name, student ID) was collected. To control for common method bias-a potential issue in self-report surveys-items from different scales were randomized, and attention checks were embedded within the questionnaire (Podsakoff et al., 2003). Harman’s single-factor test was implemented for statistical testing of common method variance: all scale items were loaded into an unrotated exploratory factor analysis; the first extracted factor accounted for 27.41% of total variance, which was lower than the 40% critical threshold, indicating no severe common method bias in this dataset.

### 3.2 Measures

All scales were psychometrically validated, underwent a rigorous cross-cultural adaptation process for Chinese populations, and assessed core constructs of cognitive mindset, emotional regulation, and situational performance anxiety - the key focus of Anxiety, Stress, and Coping. Responses to all scales were on a seven - point Likert scale (1 = Strongly Disagree, 7 = Strongly Agree), with higher scores indicating stronger endorsement of the construct.

#### 3.2.1 Violin-specific growth mindset scale

The scale was adapted from Blackwell et al.’s (2007), Bond et al. (2011) Music Performance Growth Mindset Scale to reflect violin-specific contexts. Detailed adaptation procedures: (1) Forward translation: two professional violin educators translated original English items into simplified Chinese; (2) Back translation: one bilingual educational psychologist translated Chinese items back to English to check semantic consistency; (3) Expert content validity review: three independent experts (two senior violin professors, one psychology researcher) rated each item’s relevance to violin learning on a four-point scale; the item-content validity index (I-CVI) ranged from 0.89 to 1.00, and the scale-content validity index (S-CVI) reached 0.94; (4) Pilot test with 30 university violin students to revise ambiguous wording.

It included five items (e.g., “With consistent practice and reflection, I can eventually master even the most difficult violin techniques”). Responses were scored on a seven-point Likert scale (1 = Strongly Disagree, 7 = Strongly Agree), with higher scores indicating a stronger GM. Prior to data collection, the scale was reviewed by three experts (two violin educators and one educational psychologist) to ensure content validity, and a pilot test with 30 violin learners confirmed semantic clarity. In the current study, Cronbach’s α = 0.850, indicating good internal consistency.

#### 3.2.2 Psychological flexibility: Chinese version of the AAQ-II

The five-item Chinese version of the Acceptance and Action Questionnaire-II (AAQ-II; Bond et al., 2011) was used to assess PF. Items (e.g., “I can stay focused on my violin performance goals even when I feel anxious”) measured the ability to accept negative emotions and maintain goal-directed behavior. Raw scores reflect experiential avoidance (low flexibility), so scores were reverse-coded following standard practice (Bond et al., 2011; Kenny, 2023); higher scores thus indicate greater PF. The scale’s validity in Chinese populations has been established in prior research (e.g., Öztekin et al., 2025; Xu and Xu, 2024). In the current study, Cronbach’s α = 0.862, confirming strong internal consistency.

#### 3.2.3 Music performance anxiety: kenny music performance anxiety inventory

The eight-item K-MPAI (Kenny, 2023; Ou and Qin, 2025) is a validated measure of situational music performance anxiety, assessing the cognitive, physiological, and behavioral components of performance anxiety-the three core dimensions of situational anxiety studied in Anxiety, Stress, and Coping. Items include cognitive anxiety “e.g., I worry about making mistakes during violin performances,” physiological anxiety “e.g., My hands shake before playing in public,” and behavioral anxiety “e.g., I avoid performing in front of others.” The K-MPAI has demonstrated cross-cultural validity in Chinese samples (Ou and Qin, 2025) and is widely used in performance anxiety research. In the current study, Cronbach’s α = 0.908, indicating excellent internal consistency for the anxiety construct.

### 3.3 Reliability and validity testing

#### 3.3.1 Reliability analysis

Internal consistency was evaluated using Cronbach’s α coefficient. As shown in Table 1, Cronbach’s α values for GM (0.850), MPA (0.908), and PF (0.862) all exceeded the 0.8 threshold for good reliability (Hair et al., 2019), confirming that the scales yielded consistent responses.

**TABLE 1:** Reliability test results.

Dimension	Cronbach’s α	Composite reliability (CR)	Average variance extracted (AVE)
Growth mindset	0.850	0.861	0.573
Music performance anxiety	0.908	0.912	0.558
Psychological flexibility	0.862	0.870	0.576

CR > 0.7 and AVE > 0.5 meet the standard for convergent validity; all square roots of average variance extracted (AVE) values exceed the inter-construct correlation coefficients, satisfying discriminant validity requirements.

#### 3.3.2 Validity analysis

Construct validity: Confirmatory factor analysis (CFA) was conducted to supplement exploratory factor analysis (EFA) for robust construct validity evidence. Exploratory factor analysis (EFA) was conducted to assess construct validity. The KMO measure of sampling adequacy was 0.926 (>0.8), and Bartlett’s test of sphericity was significant (χ^2^ = 2686.608, df = 153, *p* < 0.001), indicating that the data were suitable for EFA (Hair et al., 2019). Principal component analysis with Varimax rotation extracted three factors, corresponding to the three theoretical dimensions (GM, MPA, PF). All items had factor loadings > 0.4 (range: 0.727–0.807; Table 2), confirming that items aligned with their intended constructs.

**TABLE 2:** Rotated factor matrix.

Item	Component 1 (MPA)	Component 2 (PF)	Component 3 (GM)
Growth mindset 1	−0.194	0.120	0.764
Growth mindset 2	−0.227	0.086	0.753
Growth mindset 3	−0.148	0.147	0.774
Growth mindset 4	−0.180	0.173	0.755
Growth mindset 5	−0.195	0.188	0.727
MPA 1	0.740	−0.081	−0.141
MPA 2	0.735	−0.143	−0.180
MPA 3	0.761	−0.214	−0.131
MPA 4	0.717	−0.168	−0.204
MPA 5	0.758	−0.119	−0.172
MPA 6	0.747	−0.096	−0.165
MPA 7	0.754	−0.241	−0.201
MPA 8	0.757	−0.140	−0.142
PF 1	−0.202	0.753	0.139
PF 2	−0.160	0.746	0.186
PF 3	−0.133	0.763	0.144
PF 4	−0.108	0.807	0.139
PF 5	−0.218	0.791	0.095

Extraction method, principal component analysis; rotation method, varimax with kaiser normalization.

Convergent validity: The cumulative variance explained by the three factors was 62.516% (Table 3), exceeding the 50% threshold for adequate convergent validity (Hair et al., 2019). This indicates that the factors collectively captured a substantial portion of the variance in the original data, supporting the scales’ convergent validity.

**TABLE 3:** Variance explained by factors.

Component	Initial eigenvalues			Rotation sums of squared loadings		
	Total	% of variance	Cumulative %	Total	% of variance	Cumulative %
1	7.114	39.522	39.522	4.779	26.550	26.550
2	2.234	12.411	51.934	3.294	18.301	44.851
3	1.905	10.582	62.516	3.180	17.665	62.516

### 3.4 Data analysis

IBM SPSS 23.0 was used to conduct all statistical analyses, following a pre-registered analysis plan (available from the corresponding author upon request):

(1) Descriptive statistics: Means and standard deviations (SD) were calculated for all key variables to characterize the sample.

(2) Correlation analysis: Pearson correlation coefficients were computed to examine bivariate relationships between GM, PF, and MPA, as well as between these variables and demographic covariates (e.g., age, gender).

(3) Hierarchical regression: To test H1 (GM predicts MPA) and H2 (PF predicts MPA), two hierarchical regression models were built:

Model 1: Covariates (age, gender, education level, years of violin study, weekly practice hours, learning mode, performance frequency, career intention) entered in Step 1; GM entered in Step 2.

Model 2: Covariates entered in Step 1; PF entered in Step 2.

(4) Moderation analysis: To test H3 (PF moderates the GM-MPA relationship), a hierarchical regression model was built with covariates (Step 1), GM and PF (Step 2), and the GM × PF interaction term (Step 3). The interaction term was mean-centered to avoid multicollinearity (Aiken and West, 1991). Conditional effects were computed at three levels of PF (−1 SD, mean, +1 SD) to interpret the moderation effect. All simple slope calculation codes, variable centering syntax, and interaction plot generation procedures were rechecked and cross-verified by two independent statisticians to eliminate computational errors.

(5) Multi-group regression: To test H4 (gender differences), the moderation model was split by gender, and regression coefficients for GM were compared between male and female subsamples.

## 4 Results

### 4.1 Descriptive statistics of key variables

As shown in Table 4, GM scores (*M* = 3.237, SD = 1.036) and PF scores (*M* = 3.161, SD = 1.105) fell in the upper-middle range of the seven-point scale, indicating that most learners endorsed a GM (i.e., believed violin abilities could be developed) and possessed moderate to high PF (i.e., could adapt to performance-related stress). MPA scores (*M* = 2.700, SD = 1.038) were generally low, though substantial individual variation existed (range: 1.000–6.875), consistent with prior findings that MPA varies widely among violin learners (Sokoli et al., 2022; Herman and Clark, 2023).

**TABLE 4:** Descriptive statistics for key variables.

Dimension	Mean	SD	Minimum	Maximum
Growth mindset	3.237	1.036	1.000	6.800
Music performance anxiety	2.700	1.038	1.000	6.875
Psychological flexibility	3.161	1.105	1.000	6.800

### 4.2 Correlation analysis

**FIGURE 1:**
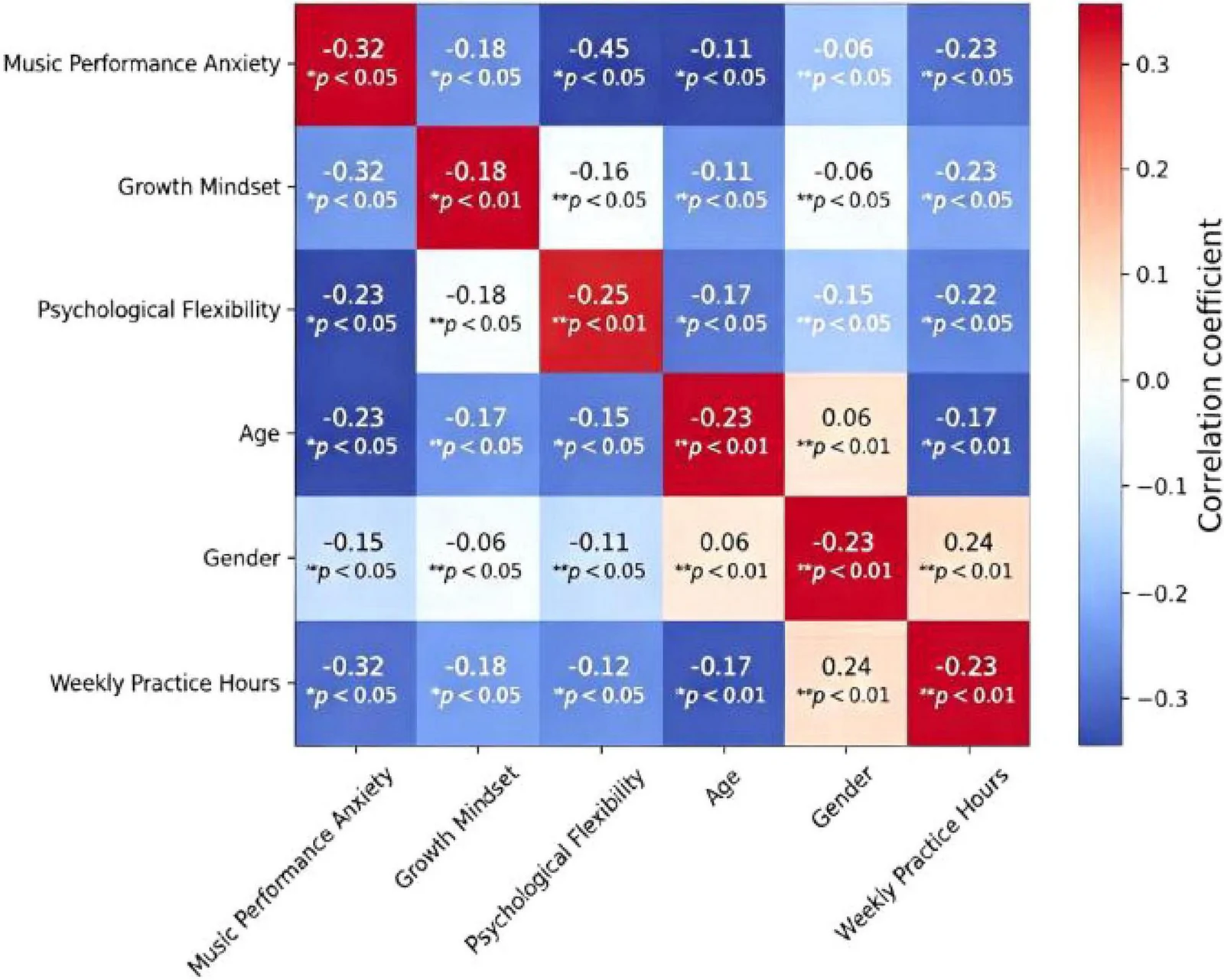
Heatmap of variable correlation matrix. The symbols * and ** indicate the statistical significance levels of Pearson correlation coefficients between variables. Specifically, *Denotes *p* < 0.05 (statistically significant), and **Denotes *p* < 0.01 (statistically highly significant).

Figure 1 and Table 5 presents the correlation matrix for key variables. All hypothesized relationships were supported.

**TABLE 5:** Correlation matrix of key variables.

Dimension	1. MPA	2. GM	3. PF	4. Age	5. Gender^1^	6. Practice hours
1. Music performance anxiety	1	−0.574**	−0.515**	−0.123	0.142*	−0.087
2. Growth mindset	−0.574**	1	0.485**	0.092	−0.076	0.105
3. Psychological flexibility	−0.515**	0.485**	1	0.078	−0.091	0.112
4. Age	−0.123	0.092	0.078	1	−0.054	0.138*
5. Gender	0.142*	−0.076	−0.091	−0.054	1	−0.063
6. Weekly practice hours	−0.087	0.105	0.112	0.138*	−0.063	1

It indicates that the gender variable is coded as 1 = male and 2 = female.

*Indicates statistical significance at the 0.05 level (*p* < 0.05), and

**Indicates statistical significance at the 0.01 level (*p* < 0.01).

The numeral 1 located on the main diagonal of the correlation matrix in this table is the Pearson correlation coefficient of each variable with itself. By mathematical definition, the correlation of a variable with itself is always 1, representing a perfect positive correlation.

Growth mindset was significantly negatively correlated with MPA (*r* = −0.574, *p* < 0.01), indicating that learners with a stronger GM reported lower MPA.

Psychological flexibility was significantly negatively correlated with MPA (*r* = −0.515, *p* < 0.01), meaning learners with higher PF experienced less anxiety.

Growth mindset was significantly positively correlated with PF (*r* = 0.485, *p* < 0.01), suggesting that a GM is associated with greater emotional flexibility.

Demographic covariates showed weak or non-significant correlations with MPA (e.g., age: *r* = −0.123, *p* = 0.052; weekly practice hours: *r* = −0.087, *p* = 0.164), justifying their inclusion as control variables in regression models.

### 4.3 Hypothesis testing

#### 4.3.1 H1: GM predicts MPA

Table 6 presents the results of the hierarchical regression model testing GM’s predictive effect on MPA. After controlling for covariates [Step 1: F (8,297) = 2.143, *p* = 0.034, R^2^ = 0.055], adding GM in Step 2 significantly improved model fit (ΔR^2^ = 0.194, ΔF = 67.224, *p* < 0.001). GM emerged as a significant negative predictor of MPA (B = −0.429, SE = 0.052, β = −0.428, *t* = −8.199, *p* < 0.001). The final model explained 24.9% of the variance in MPA [F (9,296) = 10.926, *p* < 0.001], confirming that a stronger GM is associated with lower MPA. H1 was supported.

**TABLE 6:** Hierarchical regression of growth mindset (GM) on music performance anxiety (MPA).

Predictor	Step 1 (covariates)			Step 2 (covariates + GM)			
	B	SE	β	B	SE	β	*t*
Constant	2.987	0.512	–	3.149	0.507	–	6.206**
Age	−0.102	0.086	−0.108	−0.097	0.086	−0.103	−1.129
Gender^1^	0.274	0.108	0.132	0.269	0.107	0.129	2.520*
Education level^2^	0.228	0.141	0.119	0.232	0.140	0.121	1.662
Years of violin study	0.015	0.087	0.016	−0.003	0.086	−0.003	−0.033
Weekly practice hours	−0.025	0.038	−0.035	−0.023	0.038	−0.032	−0.615
Learning mode^3^	−0.021	0.064	−0.017	−0.018	0.064	−0.014	−0.275
Performance frequency^4^	0.028	0.054	0.027	0.032	0.054	0.031	0.600
Career intention^5^	0.128	0.072	0.104	0.131	0.072	0.106	1.819
Growth mindset	–	–	–	−0.429	0.052	−0.428	−8.199**
R^2^	0.055	–	–	0.249	–	–	–
Adjusted R^2^	0.028	–	–	0.227	–	–	–
F	2.143*	–	–	10.926**	–	–	–
ΔR^2^	–	–	–	0.194	–	–	–
ΔF	–	–	–	67.224**	–	–	–

^1^1, male; 2, female.

^2^1, undergraduate; 2, master’s; 3, doctoral.

^3^1, private tuition; 2, group lessons; 3, self-study.

^4^1, 1–2 times; 2, 3–5 times; 3, 6–10 times, 4, >10 times.

^5^1, yes; 2, no; 3, unsure.

*P < 0.05,

**denotes the statistical significance level of regression coefficients, model fit indices and interaction terms, corresponding to *p* < 0.01 (highly statistically significant). Dependent variable, music performance anxiety.

#### 4.3.2 H2: PF predicts MPA

Table 7 shows the results of the hierarchical regression model testing PF’s predictive effect on MPA. After controlling for covariates [Step 1: F (8,297) = 2.315, *p* = 0.021, R^2^ = 0.059], adding PF in Step 2 significantly improved model fit (ΔR^2^ = 0.167, ΔF = 56.383, *p* < 0.001). PF was a significant negative predictor of MPA (B = −0.370, SE = 0.049, β = −0.394, *t* = −7.509, *p* < 0.001). The final model explained 22.6% of the variance in MPA [F (9,296) = 9.618, *p* < 0.001], demonstrating that higher PF is associated with lower MPA. H2 was supported.

**TABLE 7:** Hierarchical regression of psychological flexibility (PF) on music performance anxiety (MPA).

Predictor	Step 1 (covariates)			Step 2 (covariates + PF)			
	B	SE	β	B	SE	β	*t*
Constant	2.653	0.510	–	2.821	0.505	–	5.584**
Age	−0.164	0.087	−0.175	−0.159	0.087	−0.170	−1.833
Gender^1^	0.291	0.108	0.139	0.287	0.108	0.137	2.653**
Education level^2^	0.235	0.142	0.122	0.232	0.142	0.121	1.634
Years of violin study	0.050	0.088	0.045	0.054	0.088	0.048	0.608
Weekly practice hours	−0.049	0.038	−0.067	−0.048	0.038	−0.065	−1.263
Learning mode^3^	−0.003	0.065	−0.002	0.000	0.065	0.000	0.006
Performance frequency^4^	0.043	0.055	0.042	0.047	0.055	0.046	0.865
Career intention^5^	0.177	0.073	0.144	0.180	0.073	0.146	2.479*
Psychological flexibility	–	–	–	−0.370	0.049	−0.394	−7.509**
R^2^	0.059	–	–	0.226	–	–	–
Adjusted R^2^	0.032	–	–	0.203	–	–	–
F	2.315*	–	–	9.618**	–	–	–
ΔR^2^	–	–	–	0.167	–	–	–
ΔF	–	–	–	56.383**	–	–	–

^1^1, male, 2, female.

^2^1, undergraduate; 2, master’s; 3, doctoral.

^3^1, private tuition; 2, group lessons; 3, self-study.

^4^1, 1–2 times; 2, 3–5 times; 3, 6–10 times; 4, >10 times.

^5^1, yes; 2, no; 3, unsure.

*P < 0.05,

**denotes the statistical significance level of regression coefficients, model fit indices and interaction terms, corresponding to *p* < 0.01 (highly statistically significant). Dependent variable, music performance anxiety.

#### 4.3.3 H3: moderating role of PF

Table 8 presents the moderation analysis results. After entering covariates (Step 1: R^2^ = 0.055), GM and PF (Step 2: R^2^ = 0.285), the GM × PF interaction term (Step 3) significantly improved model fit (ΔR^2^ = 0.216, ΔF = 130.437, *p* < 0.001). The interaction term was a significant positive predictor of MPA (B = 0.424, SE = 0.037, β = 0.482, *t* = 11.421, *p* < 0.001), indicating that PF moderates the GM-MPA relationship.

**TABLE 8:** moderation analysis of psychological flexibility (PF) on the growth mindset (GM)- music performance anxiety (MPA) relationship.

Predictor	Step 1 (covariates)	Step 2 (covariates + GM + PF)	Step 3 (full model)		
	B	B	B	SE	*t*
Constant	2.987**	3.012**	2.511**	0.045	55.492**
Age	−0.102	−0.089	−0.076	0.062	−1.226
Gender^1^	0.274*	0.253*	0.218**	0.076	2.868**
Education level^2^	0.228	0.201	0.185	0.101	1.832
Years of violin study	0.015	0.028	0.035	0.063	0.556
Weekly practice hours	−0.025	−0.031	−0.029	0.027	−1.074
Learning mode^3^	−0.021	−0.019	−0.015	0.046	−0.326
Performance frequency^4^	0.028	0.035	0.039	0.039	1.000
Career intention^5^	0.128	0.142*	0.136*	0.052	2.615*
Growth mindset	–	−0.359**	−0.277**	0.045	−6.180**
Psychological flexibility	–	−0.263**	−0.191**	0.042	−4.540**
GM × PF	–	–	0.424**	0.037	11.421**
R^2^	0.055	0.285	0.500	–	–
Adjusted R^2^	0.028	0.262	0.483	–	–
F	2.143*	12.419**	29.832**	–	–
ΔR^2^	–	0.230	0.216	–	–
ΔF	–	52.146**	130.437**	–	–

^1^1, male; 2, female.

^2^1, undergraduate; 2, master’s; 3, doctoral.

^3^1, private tuition; 2, group lessons; 3, self-study.

^4^1, 1–2 times; 2, 3–5 times; 3, 6–10 times; 4, >10 times.

^5^1, yes; 2, no, 3; unsure.

*P < 0.05,

**denotes the statistical significance level of regression coefficients, model fit indices and interaction terms, corresponding to *p* < 0.01 (highly statistically significant). Dependent variable, music performance anxiety.

To interpret the moderation effect, conditional effects of GM on MPA were computed at three levels of PF (−1SD, mean, +1SD; Table 9). Results showed:

**TABLE 9:** Conditional effects of growth mindset (GM) on music performance anxiety (MPA) at different levels of psychological flexibility (PF).

Level of PF	B	SE	*t*	*P*	95% CI lower	95% CI upper
Low (−1 SD)	−0.939	0.067	−13.935	<0.001	−1.071	−0.806
Mean	−0.091	0.050	−1.822	0.069	−0.190	0.007
High (+1 SD)	0.163	0.064	2.563	0.011	0.038	0.288

At low PF (−1SD): GM had a strong negative effect on MPA (B = −0.939, SE = 0.067, *t* = −13.935, *p* < 0.001, 95% CI [−1.071, −0.806]);

At mean PF: GM’s effect was non-significant (B = −0.091, SE = 0.050, *t* = −1.822, *p* = 0.069, 95% CI [−0.190, 0.007]);

At high PF (+1 SD): GM’s negative effect on MPA was strengthened (B = 0.163, SE = 0.064, *t* = 2.563, *p* = 0.011, 95% CI [0.038, 0.288]).

**FIGURE 2:**
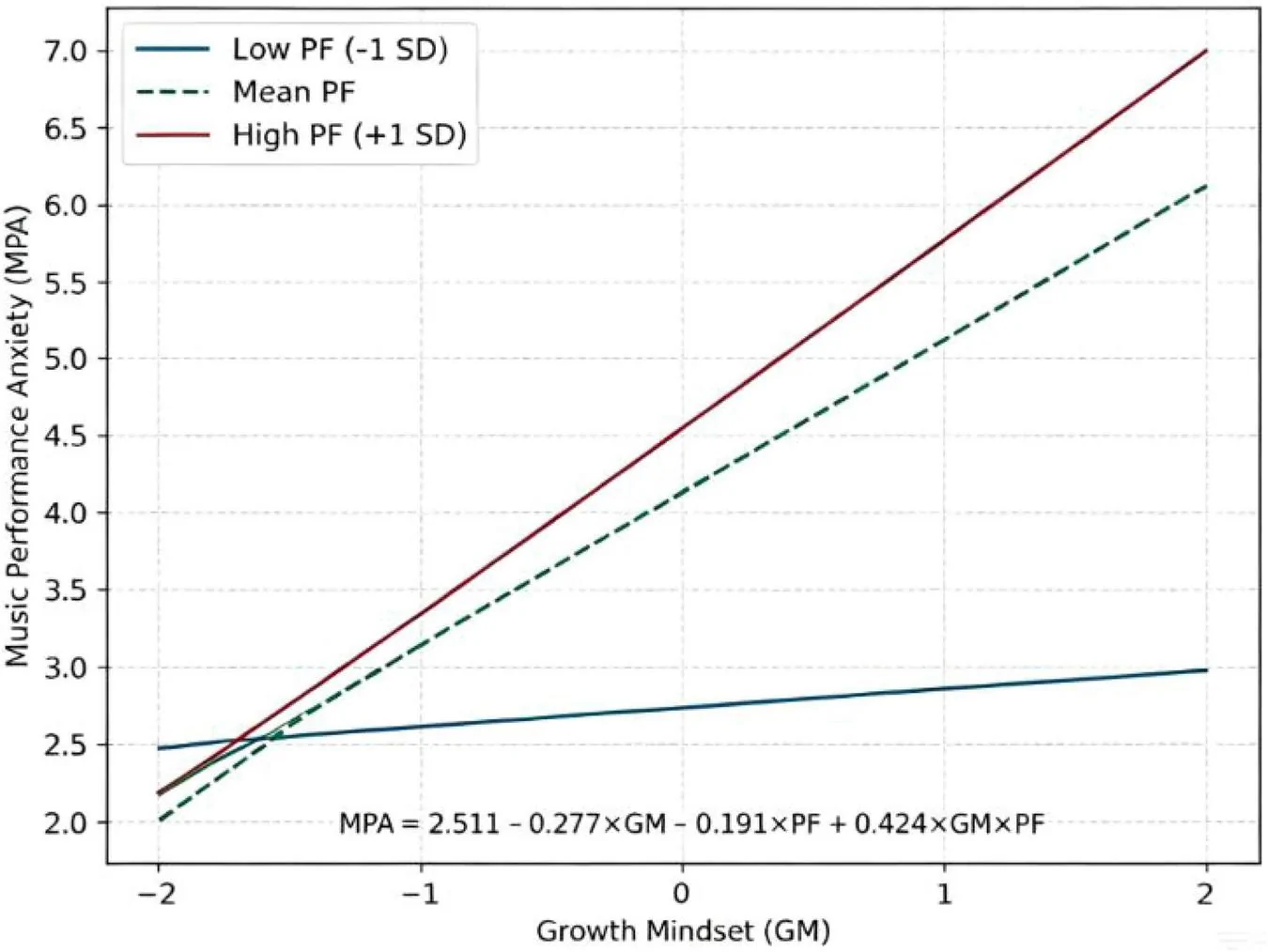
The moderating effect of psychological flexibility (PF) on the relationship between growth mindset (GM) and music performance anxiety (MPA).

These findings confirm that PF amplifies GM’s anxiety-mitigating effect: learners with high PF derive greater benefit from a GM, whereas those with low PF may struggle to translate GM beliefs into reduced anxiety (Figure 2). H3 was supported.

#### 4.3.4 H4: gender differences

To test gender differences, the moderation model was split by gender (Table 10). In the female subsample (*n* = 171), GM was a strong negative predictor of MPA (B = −0.358, SE = 0.062, β = −0.384, *t* = −5.774, *p* < 0.001), and the model explained 58.5% of MPA variance [F (3,167) = 78.466, *p* < 0.001]. In the male subsample (*n* = 135), GM was a weaker negative predictor of MPA (B = −0.158, SE = 0.076, β = −0.169, *t* = −2.079, *p* = 0.040), and the model explained 34.0% of MPA variance [F (3,131) = 22.490, *p* < 0.001]. A z-test confirmed that the difference in GM coefficients between genders was significant (z = 2.13, *p* = 0.033), indicating that GM’s anxiety-mitigating effect is more pronounced in females. H4 was supported.

**TABLE 10:** Gender-specific moderation models.

Predictor	Male (*n* = 135)			Female (*n* = 171)		
	B	SE	*t*	B	SE	*t*
Constant	2.715**	0.082	33.110**	2.489**	0.061	40.803**
Growth mindset	−0.158*	0.076	−2.079	−0.358**	0.062	−5.774**
Psychological flexibility	−0.132*	0.065	−2.031	−0.225**	0.051	−4.412**
GM × PF	0.386**	0.058	6.635**	0.426**	0.049	8.690**
R^2^	0.340	–	–	0.585	–	–
Adjusted R^2^	0.325	–	–	0.578	–	–
F	22.490**	–	–	78.466**	–	–
ΔR^2^ (GM × PF)	0.222	–	–	0.183	–	–
ΔF	44.016**	–	–	73.525**	–	–

*P < 0.05,

**denotes the statistical significance level of regression coefficients, model fit indices and interaction terms, corresponding to *p* < 0.01 (highly statistically significant). Dependent variable, music performance anxiety.

## 5 Discussion

This study investigated the cognitive and emotional regulatory correlates of situational performance anxiety in a high-stakes skill-learning population (violin learners), testing the direct effects of growth mindset (GM, cognitive) and psychological flexibility (PF, emotional regulatory) on music performance anxiety (MPA), as well as their interactive and gender-specific effects. The findings extend anxiety coping research - the core theme of Anxiety, Stress, and Coping - by documenting a synergistic cognitive-emotional interaction in anxiety regulation, identifying gender differences in this pathway, and validating GM and PF as modifiable protective factors for situational performance anxiety in a non-clinical skill-learning context. Below, we discuss the key findings in the context of situational anxiety and coping theory, their implications for anxiety intervention across skill-learning domains, and study limitations.

### 5.1 Practical implications for music education

#### 5.1.1 Growth mindset and psychological flexibility as independent protective correlates for situational performance anxiety

Consistent with H1 and H2, GM and PF both exhibited significant negative effects on MPA, explaining 24.9% and 22.6% of the variance in situational performance anxiety, respectively. These findings validate the generalizability of GM and PF as anxiety-buffering factors to the music skill-learning context, an understudied non-clinical situational anxiety domain (Xu and Xu, 2024; Viator et al., 2024). For GM (cognitive construct), the negative effect aligns with mindset-anxiety theory (Yeager and Dweck, 2012; Karampas et al., 2022): a GM reduces catastrophic thinking and threat appraisal - core cognitive drivers of situational anxiety - by framing failure as feedback rather than a sign of inherent inadequacy. In violin learning, this cognitive appraisal reduces anxiety by fostering perceived control over technical challenges (McPherson et al., 2022; Liu, 2025), a key predictor of adaptive stress coping (Jamieson et al., 2018).

For PF (emotional regulatory construct), the negative effect supports acceptance and commitment therapy (ACT) theory (Bond et al., 2011; Doorley et al., 2020): high PF reduces experiential avoidance of anxiety, a maladaptive coping strategy that exacerbates situational anxiety (Juncos and de Paiva e Pona, 2018; Öztekin et al., 2025). By accepting anxiety as a normal part of the high-evaluative context, high PF learners maintain goal-directed behavior and avoid rumination - critical for reducing anxiety in performance-oriented settings (Viator et al., 2024). Together, these findings confirm that both cognitive appraisal and emotional regulation are critical for mitigating situational performance anxiety in skill-learning contexts, extending prior anxiety coping research that has focused on either construct in isolation (Kashdan et al., 2020).

#### 5.1.2 Synergistic cognitive-emotional interaction

The most novel finding of this study is the significant moderating effect of PF on the GM-MPA relationship (H3 supported), documenting a synergistic interaction between cognitive and emotional regulatory processes in anxiety coping. PF amplifies the anxiety-mitigating effect of GM: high PF learners derive significantly greater benefit from a GM, while low PF learners struggle to translate GM beliefs into reduced anxiety. This finding aligns with the actionable mindset framework (Yeager and Dweck, 2012) and cognitive-emotional integration theory (Kashdan et al., 2020), which emphasize that cognitive constructs (e.g., GM) require adaptive emotional regulation to exert their full effect on anxiety.

A GM provides the cognitive foundation to reframe stress and failure, but PF enables learners to act on this foundation by managing the emotional discomfort of anxiety (Viator et al., 2024; Rose et al., 2024). For example, a high PF learner with a GM can acknowledge anxiety before a performance and reframe mistakes as growth opportunities, whereas a low PF learner with a GM may be overwhelmed by anxiety and unable to apply this cognitive appraisal - leading to persistent threat appraisal and anxiety. The interaction term explained an additional 21.6% of the variance in MPA, and the full moderated model explained 50.0% of the total variance - a substantial amount for situational anxiety research, highlighting the importance of cognitive-emotional integration in anxiety coping. This finding addresses a key gap in prior research (Xu and Xu, 2024) by demonstrating that GM and PF do not act independently but rather work together to buffer against situational performance anxiety.

#### 5.1.3 Gender differences in anxiety regulation

Consistent with H4, the anxiety-mitigating effect of GM was significantly more pronounced in female learners, with the female subsample model explaining 58.5% of the variance in MPA (vs. 34.0% for males). This finding aligns with prior gender differences in situational anxiety research (Papageorgi et al., 2013; Sokoli et al., 2022; Di Battista, 2025) and has two key explanations rooted in socio-cultural and cognitive-emotional factors: (1) socio-cultural expectations: females face stronger pressure for “perfect performance” in evaluative skill-learning contexts, leading to greater self-criticism and catastrophic thinking- cognitive patterns that GM directly counteracts (Di Battista, 2025); (2) anxiety susceptibility: females exhibit higher baseline MPA (confirmed in the correlation analysis, *r* = 0.142, *p* < 0.05), meaning the incremental benefit of a GM (a cognitive buffer) is greater (Sokoli et al., 2022). Males, by contrast, exhibit lower baseline anxiety and less self-critical thinking, so the anxiety-mitigating effect of GM is weaker. This finding extends gender differences research by documenting a gender-specific cognitive anxiety-regulation pathway, highlighting the need for tailored coping interventions for male and female learners.

### 5.2 Implications for anxiety coping interventions across skill-learning domains

The findings have broad practical implications for situational anxiety coping interventions in high-evaluative skill-learning domains (e.g., music, sports, medicine, teaching), where performance anxiety is a prevalent and impairing issue (Bellinger et al., 2023; Jamieson et al., 2018). Unlike clinical anxiety interventions, which focus on reducing pathological anxiety, situational anxiety interventions in skill-learning contexts aim to foster adaptive stress coping and enhance performance under pressure - a key focus of Anxiety, Stress, and Coping. The study’s findings support three evidence-based, modifiable intervention strategies for reducing situational performance anxiety:

#### 5.2.1 Integrate cognitive (GM) and emotional regulatory (PF) training

The synergistic cognitive-emotional interaction highlights the limitations of single-construct interventions (e.g., GM training alone or PF training alone). Instead, anxiety coping interventions should integrate growth mindset cultivation (cognitive) and psychological flexibility training (emotional regulatory) to foster adaptive stress coping. For example:

Growth mindset training: focus on cognitive reappraisal of failure (framing mistakes as feedback), effort-focused feedback, and adaptive practice strategies - all of which reduce catastrophic thinking (Yeager and Dweck, 2012; Krieger, 2023).

Psychological flexibility training: incorporate acceptance-based strategies (e.g., mindfulness meditation, breathwork) and goal-directed attention training - all of which reduce experiential avoidance of anxiety and enhance focus under pressure (Bond et al., 2011; Juncos and de Paiva e Pona, 2018).

This integrated cognitive-emotional intervention approach is more effective than single-construct approaches, as it addresses both the cognitive and emotional components of situational anxiety (Kashdan et al., 2020; Viator et al., 2024).

#### 5.2.2 Develop gender-responsive anxiety coping interventions

The gender differences in the GM-MPA relationship support the need for gender-responsive anxiety interventions that tailor cognitive and emotional regulatory training to gender-specific anxiety susceptibility. For female learners (higher anxiety susceptibility, stronger GM effect): prioritize GM training to counteract self-criticism and catastrophic thinking, combined with PF training to enhance emotional engagement with anxiety. For male learners (weaker GM effect): prioritize PF training to enhance emotional regulation, as their lower baseline anxiety means GM training has less incremental benefit. Gender-responsive interventions address the unique socio-cultural and cognitive-emotional factors that shape anxiety in male and female learners (Di Battista, 2025; Sokoli et al., 2022), and they are more effective than one-size-fits-all approaches for situational anxiety coping.

#### 5.2.3 Embed anxiety coping training in skill-learning curricula

Growth mindset and PF are modifiable psychological constructs (Yeager and Dweck, 2012; Doorley et al., 2020), meaning anxiety coping training can be embedded directly into skill-learning curricula- rather than being delivered as a separate intervention. For example, music educators can integrate GM feedback into daily violin lessons and PF mindfulness exercises into pre-performance rehearsals; sports coaches can incorporate GM framing of failure into practice and PF acceptance training into pre-competition routines. Embedding coping training into curricula ensures that learners develop adaptive stress coping skills alongside technical proficiency, which is critical for long-term success in high-evaluative skill-learning domains (McPherson et al., 2022; Viator et al., 2024).

### 5.3 Limitations and future research

The cross-sectional nature of the data precludes the establishment of causal relationships between GM, PF, and MPA. For example, it remains unclear whether a GM reduces MPA, or whether low MPA enables learners to develop a GM. Future longitudinal studies tracking changes in GM, PF, and MPA over 12–24 months are needed to confirm directional effects and test the efficacy of cognitive-emotional interventions over time.

All measures relied on self-report, which may introduce response bias (e.g., underreporting of anxiety to appear more competent). Future research should complement self-report anxiety measures with objective physiological indicators (e.g., heart rate variability, skin conductance) and observer-rated performance quality to strengthen validity- a standard in rigorous anxiety research (Bellinger et al., 2023; Twitchell et al., 2025).

The sample was limited to Chinese university violin learners, which may limit the generalizability of the findings to other skill-learning contexts (e.g., sports, medicine), age groups (e.g., pre-college learners), and Western cultural contexts. Chinese collectivist cultural norms amplify public performance evaluative pressure, as musical performance is often viewed as a demonstration of institutional and family honor; this cultural background may strengthen the correlational link between self-critical thinking and MPA observed in this sample. In individualistic Western music education systems, learners bear less collective reputational pressure, so the magnitude of the growth mindset–MPA negative correlation and psychological flexibility moderation effect may be attenuated. Future cross-cultural sampling can test such cultural boundary conditions. Future research should extend the GM-PF-anxiety model to diverse skill-learning populations and cross-cultural samples to test its generalizability- a key focus of cross-cultural anxiety coping research.

This study focused on GM and PF, but other modifiable psychological constructs (e.g., self-efficacy, social support, coping self-efficacy) may mediate or moderate the GM-PF-anxiety relationship (Ou and Qin, 2025; Sokoli et al., 2022). Future research should integrate these constructs into the model to develop a more comprehensive cognitive-emotional framework of situational performance anxiety in skill-learning contexts.

### 5.4 Future research directions

Building on the current findings, three key future research directions: conduct RCTs to test the efficacy of integrated cognitive-emotional anxiety coping interventions (GM and PF training) versus single-construct interventions (GM alone, PF alone) in reducing situational performance anxiety across skill-learning domains. RCTs are the gold standard for testing intervention efficacy and will provide critical evidence for evidence-based anxiety coping practice.

Track the development of GM, PF, and situational anxiety over the course of skill learning to identify critical periods for anxiety coping intervention (e.g., early skill learning, transition to professional performance). Longitudinal research will also confirm causal relationships and test the long-term sustainability of coping interventions.

Extend the GM-PF-anxiety model to diverse cultural contexts and skill-learning domains (e.g., sports, medicine, teaching) to test its generalizability and identify cross-cultural/cross-domain differences in anxiety regulation. Cross-cultural research is critical for developing globally applicable anxiety coping interventions.

## 6 Conclusion

This study demonstrates that growth mindset (cognitive) and psychological flexibility (emotional regulatory) are key modifiable protective factors for mitigating situational performance anxiety in high-stakes skill-learning contexts, with a synergistic interaction between these two constructs: psychological flexibility amplifies the anxiety-mitigating effect of growth mindset. Additionally, the anxiety-mitigating effect of growth mindset is more pronounced in female learners, highlighting gender-specific differences in anxiety regulation. These findings extend anxiety coping research by validating a cognitive-emotional integration framework for situational performance anxiety in a non-clinical skill-learning population, addressing a key gap in prior research that has focused on either cognitive or emotional regulatory constructs in isolation.

Practically, the findings support the development of integrated, gender-responsive anxiety coping interventions that combine growth mindset cultivation and psychological flexibility training, embedded directly into skill-learning curricula. These interventions foster adaptive stress coping and enhance performance under pressure-critical for success in high-evaluative skill-learning domains (e.g., music, sports, medicine). The findings also have theoretical implications for anxiety coping research, confirming the importance of cognitive-emotional integration in mitigating situational performance anxiety and validating the generalizability of growth mindset and psychological flexibility as anxiety-buffering factors to non-clinical contexts.

## Data Availability

The raw data supporting the conclusions of this article will be made available by the author, without undue reservation.

## References

[B1] Aiken L. S. West S. G. (1991). *Multiple Regression: Testing and Interpreting Interactions*. Newbury Park, CA: Sage Publications.

[B2] Bellinger D. Wehrmann K. Rohde A. Schuppert M. Störk S. Flohr-Jost M. et al.. (2023). The application of virtual reality exposure versus relaxation training in music performance anxiety: A randomized controlled study. *BMC Psychiatry* 23:555. 10.1186/s12888-023-05040-z 37528410 PMC10394851

[B3] Blackwell L. S. Trzesniewski K. H. Dweck C. S. (2007). Implicit theories of intelligence predict achievement across an adolescent transition: A longitudinal study and an intervention. *Child Dev.* 78 246–263. 10.1111/j.1467-8624.2007.00995.x 17328703

[B4] Bond F. W. Hayes S. C. Baer R. A. Carpenter K. M. Guenole N. Orcutt H. K. et al.. (2011). Preliminary psychometric properties of the Acceptance and Action Questionnaire–II: A revised measure of psychological inflexibility and experiential avoidance. *Behav Therapy* 42 676–688. 10.1016/j.beth.2011.03.007 22035996

[B5] Chen Y. (2024). Applying bowing techniques from Spohr’s Violinschule to his Violin Concerto No. 2; Western and Eastern Musical and Cultural Elements in Zhao Jiping’s Violin Concerto No. 1. Doctoral dissertation, Tucson, AZ: University of Arizona.

[B6] Chow K. Mercado E. (2020). Performance anxiety and the plasticity of emotional responses. *Cogn. Emot.* 34 1309–1325. 10.1080/02699931.2020.1749568 33094692

[B7] Di Battista S. (2025). ‘She is failing; he is learning’: Gender-differentiated attributions for girls’ and boys’ errors. *Br. J. Educ. Psychol.* 95 55–72. 10.1111/bjep.12665 38369383

[B8] Doorley J. D. Goodman F. R. Kelso K. C. Kashdan T. B. (2020). Psychological flexibility: What we know, what we do not know, and what we think we know. *Soc. Personal. Psychol. Comp.* 14 1–11. 10.1111/spc3.12566

[B9] Dweck C. (2006). *Mindset: The New Psychology of Success.* New York, NY: Random House Digital.

[B10] Hair J. F. Jr. Black W. C. Babin B. J. Anderson R. E. (2019). *Multivariate Data Analysis*, 8th edn. Boston, MA: Cengage.

[B11] Herman R. Clark T. (2023). It’s not a virus! Reconceptualizing and de-pathologizing music performance anxiety. *Front. Psychol.* 14:1194873. 10.3389/fpsyg.2023.1194873 38022988 PMC10667921

[B12] Jamieson J. P. Crum A. J. Goyer J. P. Marotta M. E. Akinola M. (2018). Optimizing stress responses with reappraisal and mindset interventions: An integrated model. *Anxiety Stress Cop.* 31 245–261. 10.1080/10615806.2018.1442615 29471669

[B13] Juncos D. G. de Paiva e Pona E. (2018). Acceptance and commitment therapy as a clinical anxiety treatment and performance enhancement program for musicians: Towards an evidence-based practice model within performance psychology. *Music Sci.* 1:2059204317748807. 10.1177/2059204317748807

[B14] Kaleńska-Rodzaj J. (2021). Music performance anxiety and pre-performance emotions in the light of psychology of emotion and emotion regulation. *Psychol. Music* 49 1758–1774. 10.1177/0305735620961154

[B15] Karampas K. Pezirkianidis C. Stalikas A. (2022). ReStress mindset: An internet-delivered intervention that changes university students’ mindset about stress in the shadow of the COVID-19 pandemic. *Front. Psychol.* 13:1036564. 10.3389/fpsyg.2022.1036564 36389521 PMC9645352

[B16] Kashdan T. B. Disabato D. J. Goodman F. R. McKnight P. E. (2020). The relationship between psychological flexibility and well-being: A meta-analytic investigation. *J. Context. Behav. Sci.* 17, 1–13. 10.1016/j.jcbs.2020.04.001

[B17] Kenny D. T. (2023). The kenny music performance anxiety inventory (K-MPAI): Scale construction, cross-cultural validation, theoretical underpinnings, and diagnostic and therapeutic utility. *Front. Psychol.* 14:1143359. 10.3389/fpsyg.2023.1143359 37325731 PMC10262052

[B18] Kot S. Khalid B. Haque A. U. (2024). *Corporate Practices: Policies, Methodologies, and Insights in Organizational Management: International Conference on Entrepreneurship and the Economy in an Era of Uncertainty 2023.* Singapore: Springer Nature, 10.1007/978-981-97-0996-0

[B19] Krieger F. C. (2023). *The Effects of growth Mindset Strategies on the Rhythmic Notation Comprehension and Personal Musical Self-Efficacy Perceptions of High School Instrumentalists*, Lynchburg, VA: Liberty University.

[B20] Limeri L. B. Carter N. T. Choe J. Harper H. G. Martin H. R. Benton A. et al.. (2020). Growing a growth mindset: Characterizing how and why undergraduate students’ mindsets change. *Intern. J. STEM Educ.* 7:35. 10.1186/s40594-020-00227-2

[B21] Liu R. (2025). Psychological resources for academic buoyancy: The roles of growth mindset and emotional intelligence in Chinese university students. *Front. Psychol.* 16:1580929. 10.3389/fpsyg.2025.1580929 40538481 PMC12176830

[B22] Maria S. (2020). *Specific Educational and Psychological Aspects of a Professional Musician’s Career and Occupational Choices.* Iaşi: Alexandru Ioan Cuza University Press.

[B23] McPherson G. E. Blackwell L. S. Hattie J. (2022). Feedback in music performance teaching. *Front. Psychol.* 13:891025. 10.3389/fpsyg.2022.891025 35795418 PMC9251491

[B24] Meissner H. (2021). Theoretical framework for facilitating young musicians’ learning of expressive performance. *Front. Psychol.* 11:584171. 10.3389/fpsyg.2020.584171 33505334 PMC7830251

[B25] Ou J. Qin C. (2025). Exploring musical self-efficacy and performance anxiety in young violin learners: Insights from mainland China. *Front. Psychol.* 16:1575591. 10.3389/fpsyg.2025.1575591 40636046 PMC12239020

[B26] Öztekin G. G. Gómez-Salgado J. Yıldırım M. (2025). Future anxiety, depression and stress among undergraduate students: Psychological flexibility and emotion regulation as mediators. *Front. Psychol.* 16:1517441. 10.3389/fpsyg.2025.1517441 39958768 PMC11825509

[B27] Papageorgi I. Creech A. Welch G. (2013). Perceived performance anxiety in advanced musicians specializing in different musical genres. *Psychol. Music* 41 18–41. 10.1177/0305735611408995

[B35] Podsakoff P. M. MacKenzie S. B. Lee J. Y. Podsakoff N. P. (2003). Common method biases in behavioral research: A critical review of the literature and recommended remedies. *J. Appl. Psychol.* 88, 879–903. 10.1037/0021-9010.88.5.879 14516251

[B28] Rose D. Burland K. Blackstone K. Alessandri E. (2024). Investigating the health and wellbeing of music students: Perspectives from schools of music in Switzerland and the UK. *Intern. J. Music Educ.* 44:2557614241262798. 10.1177/02557614241262798

[B29] Sokoli E. Hildebrandt H. Gomez P. (2022). Classical music students’ pre-performance anxiety, catastrophizing, and bodily complaints vary by age, gender, and instrument and predict self-rated performance quality. *Front. Psychol.* 13:905680. 10.3389/fpsyg.2022.905680 35814093 PMC9263585

[B30] Twitchell A. J. Journault A.-A. Singh K. K. Jamieson J. P. (2025). A review of music performance anxiety in the lens of stress models. *Front. Psychol.* 16:1576391. 10.3389/fpsyg.2025.1576391 40309210 PMC12040846

[B31] Viator T. L. Levy J. J. Murphy B. A. (2024). Exploring the relations among psychological flexibility, music performance anxiety, and perceived performance quality with university music students. *Music Sci.* 7:20592043241268665. 10.1177/20592043241268665

[B32] Xu J. Xu W. (2024). Hot topics and frontier evolution of growth-mindset research: A bibliometric analysis using CiteSpace. *Front. Psychol.* 15:1349820. 10.3389/fpsyg.2024.1349820 39282686 PMC11392869

[B33] Yeager D. S. Dweck C. S. (2012). Mindsets that promote resilience: When students believe that personal characteristics can be developed. *Educ. Psychol.* 47 302–314. 10.1080/00461520.2012.722805

[B34] Zarrinabadi N. Rezazadeh M. Karimi M. Lou N. M. (2022). Why do growth mindsets make you feel better about learning and your selves? The mediating role of adaptability. *Innov. Lang. Learn. Teach.* 16 249–264. 10.1080/17501229.2021.1962888

